# Traumatic hip dislocations in children and adolescents: diagnostic challenges and the significance of MRI imaging—a multi-center study

**DOI:** 10.1007/s00068-025-02800-2

**Published:** 2025-05-06

**Authors:** Mirjam Evi Braun, Francisco Fernandez Fernandez, Lena Riha, Hagen Schmal, Peter Schmittenbecher, Dorien Schneidmueller, Christoph Strüwind, Philipp Schwerk, Sebastian Reineke, Frank Traub, Christoph Ihle, Justus Lieber, Christina Wack, Hauke Rüther, Florian Baumann, Ingo Marzi, Lewin-Caspar Busse, Ludger Tüshaus, Miriam Adrian, Florian Bergmann, Alexander Graf, Martin M. Kaiser, Oliver Loose

**Affiliations:** 1 Philipps-Universität Marburg, Marburg, Germany; 2 Department of Orthopaedics, Olgahospital, Klinikum Stuttgart, Stuttgart, Germany; 3 University of Tübingen, Tübingen, Germany; 4 Department of Orthopedic Surgery and Traumatology, Freiburg University Hospital, Albert Ludwigs University Freiburg, Freiburg, Germany; 5 Department of Orthopedic Surgery, University Hospital Odense, Odense, Denmark; 6 Department of Pediatric Surgery, Städtisches Klinikum Karlsruhe, Moltkestr. 90, Karlsruhe, Germany; 7 Department of Pediatric Trauma Surgery and Pediatric Orthopaedics, BG Unfallklinik Murnau, Professor Küntscher Str. 8, Murnau, Germany; 8 Department of Trauma Surgery, Klinikum Garmisch-Partenkirchen, Garmisch-Partenkirchen, Germany; 9 Department of Trauma Surgery BG Unfallklinik Murnau, Professor Küntscher Str. 8, Murnau, Germany; 10 Department of Pediatric Surgery, University Hospital Dresden–Technical University of Dresden, Fetscherstraße 74, Dresden, Germany; 11 Clinic for Pediatric Surgery & Pediatric Urology and Center for Children With Severe Burn Injuries, Gesundheit Nordhessen Holding AG, Kassel, Germany; 12 Department of Orthopedics and Traumatology, University Medical Center Mainz, Mainz, Germany; 13 Department of Trauma and Reconstructive Surgery, Eberhard-Karls-University Tuebingen, BG Unfallklinik, Tuebingen, Germany; 14 Department of Pediatric Surgery and Pediatric, Urology University Children‘s Hospital, Hoppe-Seyler-Strasse 1, Tübingen, Germany; 15 Center for Orthopaedics and Trauma Surgery, University Hospital Giessen and Marburg GmbH, Philipps-Universität Marburg, Baldingerstrasse Marburg, Germany; 16 Clinic for Trauma Surgery, Orthopaedics and Plastic Surgery, University Medical Center Göttingen, Göttingen, Germany; 17 Regensburg University Medical Center, Franz-Josef-Straus-Allee 11, Regensburg, Germany; 18 Department of Trauma Surgery and Orthopaedics, University Hospital, Goethe University Frankfurt, Theodor-Stern-Kai 7, Frankfurt Am Main, Germany; 19 Department of Pediatric Surgery, University Medical Center Schleswig-Holstein, Campus Lübeck, Germany; 20 Clinic for Pediatric Surgery, Faculty of Heidelberg, University Hospital Mannheim, Mannheim, Germany; 21 Department of Pediatric Surgery, Dr. Von Hauner Children’s Hospital, Ludwig-Maximilians-Universität München, Lindwurmstrasse 4, Munich, Germany; 22 Department of Trauma Medicine and Orthopaedics, Ortenau Klinikum Offenburg, Special Orthopaedic Surgery, Offenburg-Kehl, Germany; 23 Department of Pediatric Traumatology BG Trauma Center, University Hospital for Pediatric Traumatology and Pediatric Surgery Chief, Bergmannstrost Merseburger Str. 165, 06112 Halle (Saale), Germany; 24 Regensburg University Medical Center, Franz-Josef-Straus-Allee 11, 93053 Regensburg, Germany

**Keywords:** Traumatic hip dislocation, Avascular necrosis, MRI, Concomitant injuries, Children, Diagnostic algorithms

## Abstract

**Background:**

Traumatic hip dislocations in children and adolescents are rare but can lead to severe outcomes like avascular necrosis. Delayed reductions, often due to overlooked dislocations in initial imaging, pose a major risk. The variability in symptoms and emergency care challenges early diagnosis. This multi-center study evaluates diagnostic approaches to enhance protocols for identifying traumatic hip dislocations in childhood.

**Methods:**

This retrospective multi-center study included 76 patients (aged ≤ 17 years) with acute traumatic hip dislocations and open growth plates from 16 German hospitals. Patient data and imaging from 1979 to 2022 were analyzed, with statistical evaluation performed using SPSS under ethical guidelines.

**Results:**

X-rays (single and biplanar views) were the primary diagnostic method, utilized in 85% of cases. Dislocations were missed in 12% (9 cases), primarily among children under eight years, with half of those under four. Delayed reductions (15.8%, n = 12) were linked to undetected dislocations in imaging in 9 cases. Conventional X-rays frequently missed dislocations, whereas MRI successfully identified all cases. Among the 76 patients, 54 (71%) had associated injuries, with 57.9% (n = 44) diagnosed exclusively via MRI.

**Conclusion:**

Timely diagnosis of traumatic hip dislocations is crucial, as delays increase the risk of femoral head necrosis. An algorithmic approach is essential for young children, where dislocations may not be readily suspected. MRI is vital in the secondary diagnostic phase, providing superior visualization of associated injuries, including acetabular avulsions and soft tissue interpositions highlighting the need for integration of MRI into a unified diagnostic algorithm for children suspected of such injuries.

**Level of evidence:**

IV.

## Background

Traumatic hip dislocations are exceedingly rare in childhood and adolescence [2, 5, 7, 15, 16, 18, 24], but severe injuries, such as unrecognized dislocations or delayed treatment of non-concentric reductions are associated with potentially devastating consequences, like secondary coxarthrosis and avascular necrosis of the femoral head (AVN) [7, 26]. Most studies highlight delayed reductions as a common issue [2, 8, 25, 30]. For example, Offierski et al. report a dislocation diagnosed three months after trauma [21]. Barquet et al. describe 50% of initially overlooked dislocations with concurrent ipsilateral femoral shaft fractures [3]. Similarly, the previous study by this research group identifies delayed reductions in 15.8% of cases (Braun, et al., 2023). These delays are primarily being linked to overlooked dislocations through utilization of conventional radiology. It is speculated that this is due to pain-related difficulties and thus inadequate imaging [20], as well as to associated injuries in polytrauma cases [23]. Another explanation for overlooked dislocations is inherent in childhood emergency care settings, where mono-injuries often predominate [6]. Thus, primary care typically occurs not in trauma bays but in emergency departments. Primary care in "regular German emergency departments" can lead to trivialization of trauma, especially when only a minor mechanism of injury is present [30]. Furthermore, diverse mechanisms of injury, ranging from tripping in young children to high-velocity traumas in older children, further complicate the diagnosis [6, 21]. Additionally, the clinical symptomatology of patients is inconsistently described in the literature and considered unreliable for identifying hip dislocation [5, 7, 11]. Currently, conventional X-rays are established as the standard pre- and post- reduction. However, computed tomography (CT) and particularly magnetic resonance imaging (MRI) are attributed with a varied significance in the acute phase of traumatic hip dislocation [4, 24, 28]. Fabricant et al. demonstrate that ossification of the posterior acetabular wall usually occurs between twelve and thirteen years of age [10], and that conventional X-rays and CT scans may not effectively depict the non-ossified, developing, cartilaginous portions of the immature acetabulum [29]. In summary, overlooked dislocations are associated with pain and a higher risk of complications. Overlooked dislocations lead to delayed reductions, which in turn pose the greatest risk factor for developing AVN [18]. It remains to be noted that according to the literature, a multitude of factors can contribute to the overlooking of dislocations. The most easily modifiable variable appears to be that of diagnostics. Thus far, diagnostics are inconsistent, and the benefits of individual diagnostic methods, particularly imaging, have not been comparatively examined regarding childhood hip dislocation. Additionally, there is currently no diagnostic algorithm for suspected childhood traumatic hip dislocation. The aim of this multi-center study is to evaluate the individual steps of diagnostics with the goal of establishing an algorithm that improves the quality of diagnostics regarding traumatic hip dislocations in childhood.

## Materials and methods

This retrospective, anonymized data collection was conducted as a multi-center study. It includes 76 patients with acute traumatic hip dislocations. Patient recruitment was based on a nationwide request to 36 pediatric traumatology clinics through the network of the Pediatric Traumatology Section (SKT) of the German Society of Orthopedics and Trauma Surgery (DGOU). Out of 36 clinics, 16 clinics reported treating children and adolescents with traumatic hip dislocation during the period from 1979 to 2022 [6]. For easier comparability with the largest study on this topic to date [18], the inclusion criteria of this study were internalized. Patients with a history of radiologically confirmed traumatic hip dislocation and open growth plates at the proximal femur at the time of injury were included.

Patients with a positive history of hip dysplasia and association with syndromal diseases (e.g., Trisomy 21), neurological conditions (e.g., Infantile Cerebral Palsy), or connective tissue disorders (e.g., Marfan Syndrome), all of which predispose patients to hip dislocation [16], were excluded. Another exclusion criterion was insufficient data availability.

From medical records, the following information was extracted: gender, age at the time of dislocation, associated fractures, mechanism of injury, initial care including diagnostic procedures, and the time interval between dislocation and reduction. Additionally, all documented associated injuries, their diagnostic procedures, and the time interval until treatment were recorded.

**The following five age categories were established:** 1–3 years 11 months, 4 years–7 years 11 months, 8 years–10 years 11 months, 11 years–14 years 11 months, and > 15 years. This classification, established by Trueta, is based on morphological changes in the femoral head and its blood supply [27]. This categorization has already proven effective in the previous study by Braun et al. [6] and was thus continued. Additionally, the work of Yang et al. [29] supports the significance of the different morphology of age groups for imaging diagnostics.

**The clinical symptoms at initial presentation** were derived from patient records and were examined based on the characteristics listed in the literature for posterior dislocations with hip flexion, adduction, internal rotation, and shortened ipsilateral limb, as well as for anterior dislocations with hip extension, abduction, external rotation [12, 30].

**The documentation of primary imaging diagnostics** (ultrasound, conventional X-ray, MRI, or CT) as well as documentation of whether the dislocation was recognized, was extracted from patient records. The analysis included the age-dependent distribution of these imaging diagnostics as well as the age-dependent reliability of these diagnostics. Additionally, a detailed analysis of imaging was conducted using the six-eye principle to determine whether experienced pediatric traumatologists should have recognized dislocations based on the available images. This involved examining whether potential failures to recognize were attributable to imaging issues or human error.

**The reduction period** was divided into the time until the first reduction attempt, regardless of its success, and the time until the final successful reduction. These periods may differ from each other due to non-congruent reductions, which can be caused by interposition or associated injuries [26].The 6-h threshold was chosen based on the study by Mehlmann et al., which demonstrates a significantly increased risk of avascular necrosis when reduction occurred beyond 6 h. This provides a well-established benchmark for timely intervention.

**The mechanism of injury was classified into mild, moderate, and severe trauma, defined as follows**: Mild trauma included running, tripping, or falls (under three meters); moderate trauma resulted from excessive speed (riding, cycling, skiing, or sledding accidents) or excessive force (soccer); severe trauma were caused by high-velocity impact or falls (over three meters) [6]. **The type of trauma** was subdivided into isolated dislocation (mono-injuries) as well as multiple injuries and polytrauma.

**Concomitant injuries to the acetabulum were identified**. The focus was on the posterior acetabulum, as injuries in hip dislocations are almost exclusively located in this area [19]. It was analyzed whether the likelihood of associated injuries after hip dislocation is age-dependent. The associated injuries themselves were morphologically categorized based on available radiological imaging using the six-eye principle, as well as by assessment from experienced radiologists, into bony injuries, cartilage, labral, or combination injuries, as well as isolated dislocations without associated injuries. Special attention was paid to the modality for diagnosing associated injuries and the resulting diagnostic consequences.

**The diagnostic algorithm** was developed through a structured consensus-building process among the participating authors. This involved multiple rounds of discussions where the study results were critically analyzed alongside relevant literature. Each proposed step of the algorithm was debated, considering both evidence and clinical experience. The process was iterative, with continuous refinement until a consensus was reached, ensuring the algorithm was robust, evidence-based, and practical for clinical application.

**The statistical analysis** was performed using SPSS. Qualitative variables were described using absolute and relative frequencies. A bivariate analysis was conducted. Categorical variables were compared using chi-square tests (or Fisher's exact test for small datasets). Ordinal variables were compared using the linear-by-linear association test.

This study is based on the analysis of patient records and existing imaging without intervention in the treatment algorithm.

The study has received a positive ethics vote from the State Medical Association of Baden-Württemberg (F-2020-120).

## Results

**The clinical symptoms at initial presentation** could only be traced in 22.3% (n = 17) of the total 76 patients based on patient records. Among them, 19.7% (n = 15) were diagnosed with posterior dislocation. Of these, only 60% (n = 9) exhibited internal rotation, 53.3% (n = 8) exhibited flexion, 20% (n = 3) exhibited adduction, and 20% (n = 3) exhibited shortening of the ipsilateral limb, as described in the literature as typical symptoms. Two patients with posterior dislocation exhibited external rotation contrary to the literature. One patient with anterior dislocation exhibited internal rotation contrary to the clinical presentation described in the literature. The second patient with anterior dislocation exhibited only flexion.

**Delayed initial reduction** (> 6 h after trauma) occurred in 15.8% (n = 12) of the children. The reason for 12% (n = 9) was an overlooked dislocation in imaging. The cause was unknown in 5.3% (n = 3).

**Fig. 1:**
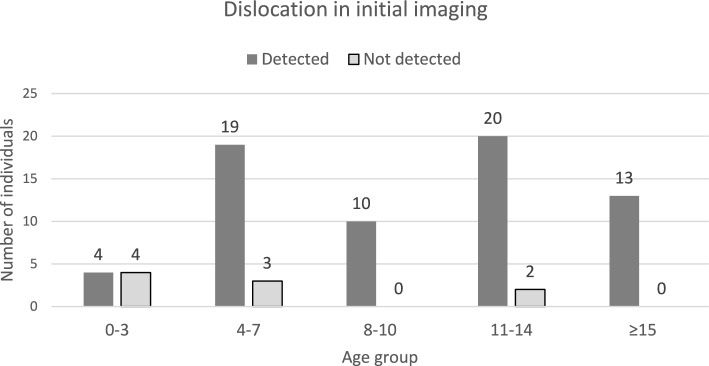
Detected dislocations in the initial imaging

**Fig. 2:**
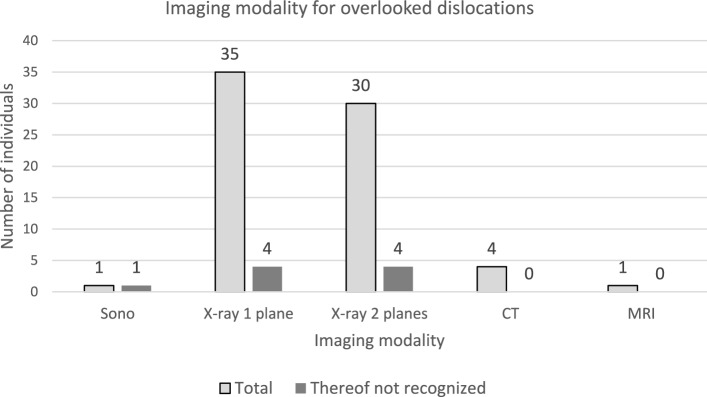
Imaging modality for overlooked dislocations

**Fig. 3:**
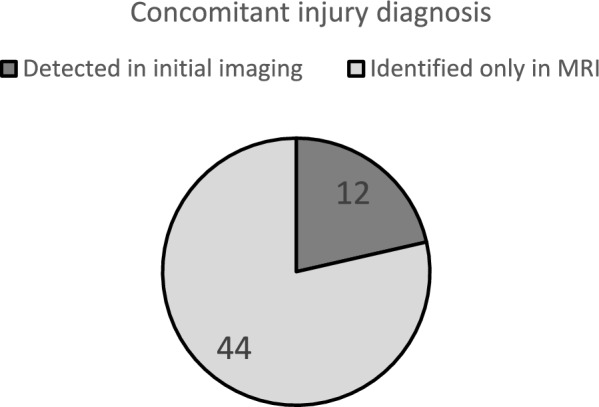
Concomitant injury diagnosis

**For primary imaging diagnostics conventional X-ray was used predominantly.** This was divided into X-rays in one plane (n = 35), biplanar X-rays (n = 30), CT (n = 4), MRI (n = 1), and ultrasound (n = 1). Although the distribution of diagnostics is seemingly very different, considering the age groups, this is not statistically significant with a p-value of 0.7704 (Table 1). Dislocation was recognized in 87% (n = 66) of primary imaging diagnostics and overlooked in 12% (n = 9). Dislocations were predominantly overlooked in younger children, with seven overlooked dislocations occurring in children under eight years old. In children under four years old, 50% of dislocations were not recognized. The differences in recognition accuracy between age groups were significant with a p-value of 0.00637 (Table 1, Fig. 1). Among cases where dislocations were not diagnosed, 44.4% (n = 4) were on conventional X-rays in one plane, 44.4% (n = 4) were on biplanar X-rays, and 11.1% (n = 1) were on ultrasound. On MRI all dislocations were detected (Figs. 2, 3).

**Table 1:** Diagnostic accuracy and missed diagnoses in traumatic hip dislocations

	Total (n/percent)	Age 0–4 Years (n/percent)	Age 4–8 years	Age 8–11 years	Age 11–15 years	Age > 15 years	p-Value
	76/ 76	(n = 10)	(n = 20)	(n = 12)	(n = 25)	(n = 9)	
		13.2%	26.3%	15.0%	32.9%	11.8%	
Primary diagnosis							p = 0.7704
Sonography	1/76	(n = 0)	(n = 0)	(n = 0)	(n = 1)	(n = 0)	
	1.4%	0.0%	0.0%	0.0%	4.0%	0.0%	
X-ray single plane	35/76	(n = 3)	(n = 10)	(n = 5)	(n = 12)	(n = 5)	
	50.0%	30.0%	52.6%	50.0%	52.2%	62.5%	
Biplanar X-ray	30/76	(n = 7)	(n = 9)	(n = 4)	(n = 8)	(n = 2)	
	42.9%	70.0%	47.4%	40.0%	34.8%	25.0%	
MRI	1/76	(n = 0)	(n = 0)	(n = 0)	(n = 1)	(n = 0)	
	1.4%	0.0%	0.0%	0.0%	4.3%	0.0%	
CT	4/76	(n = 0)	(n = 0)	(n = 1)	(n = 2)	(n = 1)	
	5.7%	0.0%	0.0%	10.0%	8.7%	12.5%	
	For five patients, no initial imaging is known or has been performed Quantity irrespective of whether the dislocation or accompanying injuries were identified						
Dislocation detected in initial imaging							p = 0.006375
Detected	66/76	(n = 4)	(n = 19)	(n = 10)	(n = 20)	(n = 13)	
	86.8%	50.0%	86.4%	90.9%	90.9%	100.0%	
Not detected	9/76	(n = 4)	(n = 3)	(n = 0)	(n = 2)	(n = 0)	
	11.8%	50.0%	13.6%	0%	(9.1%)	0.0%	
Unknown	1/76	(n = 0)	(n = 0)	(n = 1)	(n = 0)	(n = 0)	
	1.4%	0.0%	0.0%	9.1%	0.0%	0.0%	
Concomitant injuries							p = 0.04010
No	22/76	(n = 5)	(n = 9)	(n = 3)	(n = 4)	(n = 1)	
	28.9%	6,6%	11,8%	3,9%	5,3%	1,3%	
** Yes	54/76	(n = 3)	(n = 13)	(n = 8)	(n = 18)	(n = 12)	
	71.1%	3,9%	17,1%	10,5%	23,7%	15,8%	
Without injury to the joint structure	36/76	(n = 7)	(n = 15)	(n = 4)	(n = 8)	(n = 2)	P = 0.001
	%	70.0%	75.0%	36.4%	32.0%	22.2%	
Labral injury	18/76	(n = 2)	(n = 4)	(n = 4)	(n = 5)	(n = 3)	
	%	20.0%	20.0%	36.4%	20.0%	33.3%	
Cartilage and bone	5/76	(n = 0)	(n = 0)	(n = 0)	(n = 4)	(n = 1)	
	%	0.0%	0.0%	0.0%	16.0%	11.1%	
Bone	16/76	(n = 1)	(n = 1)	(n = 3)	(n = 8)	(n = 3)	
	%	10.0%	5.0%	27.3%	32.0%	33.3%	
	This also includes associated injuries outside the joint (nerve lesions, muscle hematomas, femoral head fractures, torn lig. capitis femoris, etc.)

**Fig. 4:**
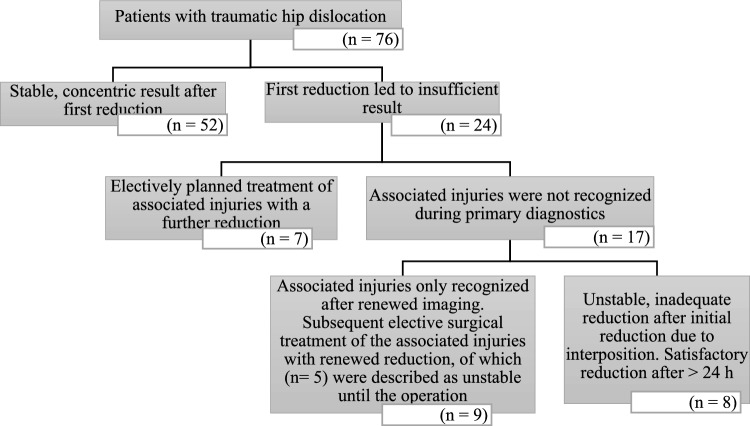
Repositioning process

**In 31.6% (n = 24), one reduction was not sufficient for final stabilization** and centering of the hip joint. In 17 cases, a missed associated injury in the primary imaging was the cause. In eight children, an interposition prevented a concentric, stable reduction. This resulted in a delayed final reduction with associated injury management, if present, in one case between 6–12 h after trauma and in 23 cases after more than 24 h after trauma (Fig. 4).

**Concomitant injuries to the acetabulum** occurred in 54 patients. With a p-value of 0.04010, the likelihood of associated injuries in younger children was significantly lower. There were significant differences in the type of associated injuries regarding age, with a p-value of 0.001. The distribution showed an increased incidence of isolated dislocations without posterior acetabulum injury in children up to eight years old. Noticeably, avulsions of the cartilaginous acetabular rim and bone injuries increased with age (Table 1). 81.5% (n = 44) of associated injuries were diagnosed by MRI (Fig. 3). In 34 cases, intervention-requiring associated injuries were identified after reduction. In 10 cases, this occurred before reduction.

## Illustrative cases

**Patient 1** fell while sledding at the age of three years and nine months. Due to severe pain and no longer being able to bear weight on the lower extremity, biplanar X-rays were taken at an external clinic on the same day. Following a suspected diagnosis of epiphysiolysis, the patient was transferred to a larger hospital. After an MRI, a diagnosis of right posterior hip dislocation was made. The subsequent closed reduction occurred more than 24 h after the trauma. Two months after the trauma, a suspicion of partial AVN was raised during an MRI follow-up.

**Fig. 5:**
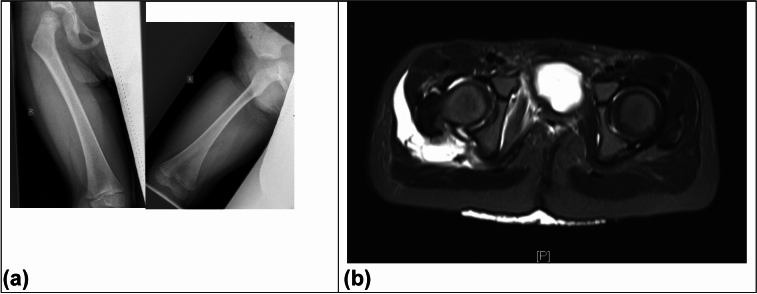
Patient 1: **a** Immediately after trauma biplanar X-ray in: Suspected epiphysiolysis; **b** > 24 h after trauma MRI after reduction

**Fig. 6:**
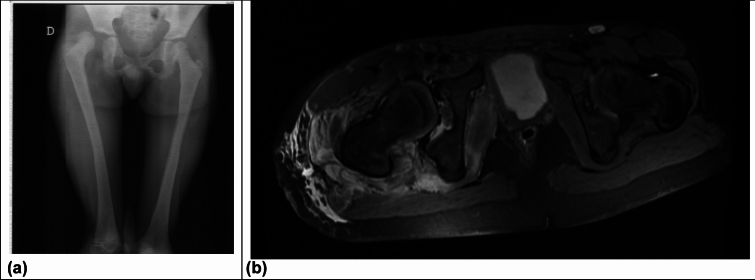
Patient 2 **a** After accident, X-ray pelvic overview, posterior dislocation; **b** Five days after accident, MRI with soft tissue interpositions

**Patient 2** slipped down a slope and rolled approximately 40 m at the age of nine years and seven months. A pelvic overview X-ray was performed at an external hospital, diagnosing a right posterior hip dislocation. Closed reduction was performed within six hours of the trauma. The hip joint was unstable after reduction. Subsequent MRI imaging revealed the interposition of soft tissue. The hip was only stabilized after two open reductions. The first was performed through a Stoppa approach one day after the trauma. The second was through a Kocher-Langenbeck approach three days after the trauma. Additionally, a paraforaminal sacral fracture was observed at an follow-up MRI. Four months after the trauma, a follow-up MRI also lead to the diagnosis of AVN (Fig. 5, 6).

## Discussion

This multicenter study serves as a continuation of the 2023 published epidemiological study on traumatic hip dislocations in childhood [6]. With 76 included patients, it currently represents the largest study on this topic to date.

The aim of this study was to evaluate diagnostic processes and it was possible to highlight significant deficits in initial diagnosis within the studied population. This reinforces existing literature documenting diagnostic challenges, where missed dislocations and delayed reductions significantly increase the risk of severe complications [3] [18]. To address the complexity of the issue, the individual steps of diagnostics were examined chronologically to develop a comprehensive diagnostic algorithm.

**Step 1. Clinical diagnosis:** Clinical symptoms for posterior dislocations are described as flexion, adduction, internal rotation, and shortening of the ipsilateral leg, and for anterior dislocations as abduction, external rotation, and hip extension [12]. These descriptions have been challenged due to their reliance on subjective factors [11]. The findings of this study only partially align with these symptoms. This could be due to accompanying injuries altering the clinical symptoms. Notably, clinical symptoms were documented in only 17 children, and few matched the clinical presentation described in the literature. While clinical examination remains undisputedly important, it should be viewed as a non-specific indication rather than a definitive diagnostic tool. Also, any abnormal position of the lower extremity should raise suspicion of hip dislocation [12]. Moreover, clinical examination of a child with a painful hip joint is challenging and thus offers limited diagnostic value. Consequently, movement testing should not be forced as it provides little diagnostic information. It is crucial to proceed immediately with diagnostic-imaging screening to allow a more precise assessment of the hip region. Based on literature and the findings of this study, clinical diagnosis is the initial step in the algorithm but should be considered non-specific.

**Step 2: Age-Based Division and the Role of Sonography: Sonography** is becoming increasingly important in trauma-oriented clinics due to its ability to be carried out quickly, cost-effectiveness, and ability to perform both static and functional diagnostics without exposing children to radiation, which is particularly crucial during growth. Sonography is a particularly effective tool in evaluating differential diagnoses like transient synovitis, the most common affliction in children's hips under the age of six to eight. Therefore, sonography plays an important role in cases of unclear hip complaints, helping to exclude differential diagnoses. However, current literature and data from this current study indicate that sonography is unsuitable as a sole primary diagnostic tool for traumatic hip dislocations in growth age (Table 1, Fig. 2). In this study, only one case involved sonography. In this one case, the dislocation was not detected. The limitations of sonography is due to two factors. Firstly, sonography is examiner-dependent and can lead to incorrect results in inexperienced hands [13]. Secondly, according to Graf et al., hip dislocations can only be reliably detected via sonography in children up to six months old due to increasing ossification. Beyond this age, sonography is limited to detecting an effusion or a hematoma [11], which may only provide indirect, unspecific indications of trauma when compared with the opposite side of the body. It should be noted that Graf et al. focus on dislocations of DDH (Developmental Dysplasia of the Hip) hips in their study. However, it is also the only study on this topic. There are experience reports suggesting sonographic assessment of traumatic hip dislocations may be possible, though confirmatory literature and statistical data are lacking. In conclusion, Step 2 divides diagnostic approaches by age: sonography is recommended for children under eight years as a non-specific tool for identifying hip dislocation and excluding differential diagnoses, while conventional X-ray is advised as the primary diagnostic method for children over eight years.

**Step 3: Ensuring Diagnostic Accuracy and Reliability:** In the current patient cohort, 15.8% (n = 12) of hip dislocations were reduced late. This is consistent with previous literature reporting delayed reductions [2, 8, 25, 30]. In 12% (n = 9) of cases, the dislocation was missed in imaging. In 4% (n = 3), the cause remained unclear. The analysis using the six-eye principle suggests that experienced pediatric traumatologists should have detected the dislocation in at least two of the nine cases. Thus, imaging represents a critical factor for timely reduction.

**In primary imaging,** conventional X-rays in one plane 46.1% (n = 35) and biplanar X-rays 39.5% (n = 30) remained the standard, while modalities like sonography, CT, or MRI only played a subordinate role to date. Overall, 12% (n = 9) of dislocations were undetected in imaging, with almost all (88.9%, n = 8) of these overlooked in conventional X-rays. It is striking that the dislocations were predominantly overlooked in children under eight years of age, in seven cases. Particularly concerning is the 50% rate of overlooked dislocations in children under four years of age.

One explanation may provide the technical limitations of X-ray diagnostics, as the bone-cartilage ratio, which only changes in favor of bone with increasing age [30]. Since conventional X-rays only assess ossified parts of the hip joint, their diagnostic value is limited in younger children. This is supported by Fabricant et al.'s study, which shows that the posterior acetabular wall ossifies more slowly than the anterior over the entire developmental period, remaining cartilaginous between ages four and eight and only ossifying from age eight onward. [10]. Consequently, the posterior wall is less stable up to this point compared to the anterior wall, which ossifies earlier. This is reflected in the results, where posterior dislocations predominate significantly during the growth period [6].

Fabricant et al. further found that the ossification of the posterior acetabular wall is typically completed by age 13, with boys experiencing a delay of 1.5 years compared to girls. These findings are consistent with previous research by Braun et al., which indicates that dislocations are more common in younger girls and older boys. Fabricant et al. go so far as to indicate that MRI is the preferred diagnostic tool for any type of hip pain in girls up to the age of 12 and in boys up to the age of 14. In this current study, no dislocations were overlooked when MRI diagnostics were applied. Thus, in Step 3, MRI should be performed whenever there is any uncertainty regarding the presence of a hip dislocation.

**Fig. 7:**
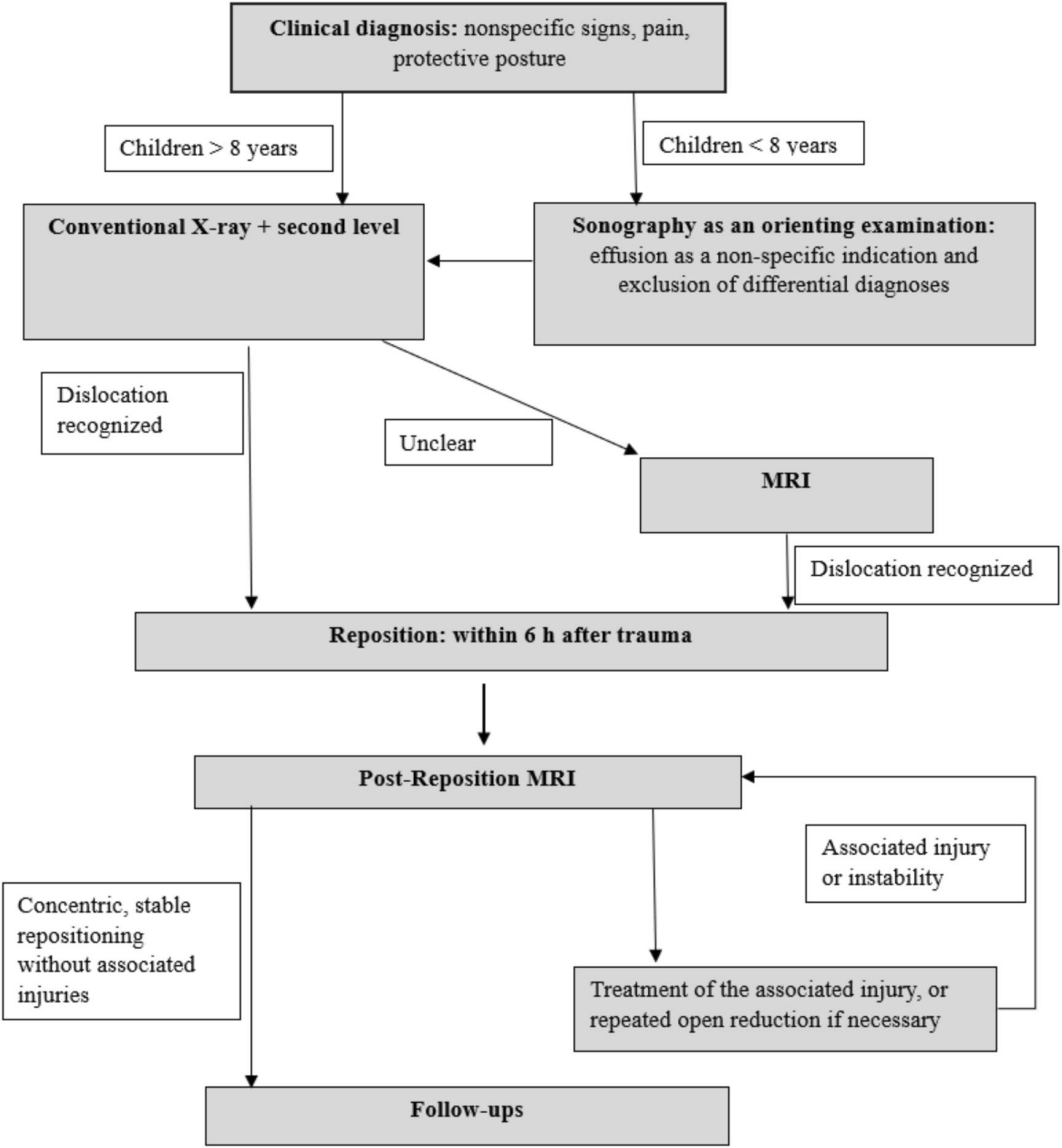
Algorithm

The algorithm depicted in Fig. 7 integrates the results of Fabricant et al. as well as the results from this study. It also takes into account the logistical effort and cost intensity that would be associated with performing an MRI on every child with hip pain. In children under four years of age, sonography is recommended to rule out differential diagnoses, followed by a pelvic overview X-ray or MRI in case if suspicion persists.

Considering the study results, the application of a second radiographic diagnostic level should be regarded as frequent with 39.5% of the cases. Worldwide, the principle of "as low as reasonably achievable" (ALARA) has been established in the assessment of pediatric fractures, aiming to minimize the radiation exposure for both the examiner and the patient [14]. Accordingly, if a clear indication for surgery can be determined on one level, a second level should only be documented during surgery under anesthesia, ensuring minimal patient discomfort [9].

**Step 4: MRI Reevaluation: In 31.6% (n = 24) of cases in this study, a single reduction was not sufficient to achieve a concentric, stable outcome.** In 17 cases, an overlooked concomitant injury in the primary imaging was causative, while interpositions prevented stable reduction in eight cases. Notably, 81.5% of concomitant injuries were diagnosed using MRI imaging. These findings align with previous studies describing capsulolabral interpositions and concomitant injuries that hinder stable reduction [17, 22, 26]. It is crucial to note that MRI proved superior in detecting both associated injuries and interpositions, as the full extent of such structures was not visualized on conventional X-rays or CT due to incomplete ossification. Therefore, due to morphological considerations, CT also does not offer an advantage over MRI [22, 24, 29]. Additionally, there is what Ashman et al. term as Satisfaction of Search in Osteoradiology. They explain that the rate of detecting additional injuries in radiographic imaging decreases with the severity of the initially detected problem.

In this study, the combination of all described factors resulted in a delayed final reduction with management of associated injuries, if present, in 1.3% (n = 1) between 6–12 h after the trauma and in 30.3% (n = 23) after more than 24 h after the trauma. To minimize complications from delayed or inadequate reductions, a reliable diagnosis before and after reduction is essential. Post-reduction MRI is therefore recommended in Step 4 in cases of instability or suspected concomitant injuries to ensure accurate assessment and timely intervention.

To establish a **diagnostic algorithm**, a thorough understanding of the developmental anatomy of the pediatric hip is crucial. Building on this foundation and the findings of this current study, a consensus-based diagnostic algorithm was developed (Fig. 7).

## Conclusion

The focus is on successful primary diagnosis of traumatic hip dislocation, which is delayed recognized in 15% of cases and carries an increased risk of avascular necrosis of the femoral head. Especially in the acute primary diagnostic phase, an algorithm is necessary to minimize the risk of being overlooked. This is particularly important for small children, where dislocation is not automatically anticipated in the emergency department due to the low energy involved at accident. Given the high incidence of missed dislocations in children up to eight years of age, an MRI should be conducted whenever there is any diagnostic doubt, especially when treating this age group.

Consequently, MRI also serves as a crucial diagnostic tool for the second diagnostic phase when considering the high incidence of concomitant injuries and should thus be added as part of the standard trauma examination process in determining and diagnosing such injuries in all children. MRI aligns significantly better with the child's anatomical conditions, especially concerning the depiction of avulsion of the cartilaginous acetabular rim and soft tissue interpositions. These factors highlight the timely and indispensable role of MRI diagnostics in traumatic pediatric hip dislocations and underscore the significance of MRI diagnostics in the presented algorithm, and should be utilized as a tool when diagnosing children with such suspected injuries.

## Data Availability

No datasets were generated or analysed during the current study.

## Abbreviations

ALARA: As low as reasonably achievable
AVN: Avascular necrosis of the femoral head
CT: Computed tomography
DDH: Developmental dysplasia of the hip
DGOU: German society of orthopedics and trauma surgery
MRI: Magnetic resonance imaging
SKT: Pediatric traumatology section
